# Urinary Untargeted Metabolic Profile Differentiates Children with Autism from Their Unaffected Siblings

**DOI:** 10.3390/metabo12090797

**Published:** 2022-08-26

**Authors:** Anna Maria Timperio, Federica Gevi, Francesca Cucinotta, Arianna Ricciardello, Laura Turriziani, Maria Luisa Scattoni, Antonio M. Persico

**Affiliations:** 1 Department of Ecological and Biological Sciences, University of Tuscia, 01100 Viterbo, Italy; 2 Interdepartmental Program “Autism 0-90”, “G. Martino” University Hospital, 98124 Messina, Italy; 3 IRCCS Centro Neurolesi “Bonino-Pulejo”, 98124 Messina, Italy; 4 Villa Miralago, 21050 Cuasso al Monte, Italy; 5 Research Coordination and Support Service, Istituto Superiore di Sanità, 00161 Rome, Italy; 6 Child & Adolescent Neuropsychiatry Program, Modena University Hospital & Department of Biomedical, Metabolic and Neural Sciences, University of Modena and Reggio Emilia, 41121 Modena, Italy

**Keywords:** metabolomics, autism, mass spectrometry, sibling

## Abstract

Autism Spectrum Disorder (ASD) encompasses a clinical spectrum of neurodevelopmental conditions that display significant heterogeneity in etiology, symptomatology, and severity. We previously compared 30 young children with idiopathic ASD and 30 unrelated typically-developing controls, detecting an imbalance in several compounds belonging mainly to the metabolism of purines, tryptophan and other amino acids, as well as compounds derived from the intestinal flora, and reduced levels of vitamins B6, B12 and folic acid. The present study describes significant urinary metabolomic differences within 14 pairs, including one child with idiopathic ASD and his/her typically-developing sibling, tightly matched by sex and age to minimize confounding factors, allowing a more reliable identification of the metabolic fingerprint related to ASD. By using a highly sensitive, accurate and unbiased approach, suitable for ensuring broad metabolite detection coverage on human urine, and by applying multivariate statistical analysis, we largely replicate our previous results, demonstrating a significant perturbation of the purine and tryptophan pathways, and further highlight abnormalities in the “phenylalanine, tyrosine and tryptophan” pathway, essentially involving increased phenylalanine and decreased tyrosine levels, as well as enhanced concentrations of bacterial degradation products, including phenylpyruvic acid, phenylacetic acid and 4-ethylphenyl-sulfate. The outcome of these within-family contrasts consolidates and extends our previous results obtained from unrelated individuals, adding further evidence that these metabolic imbalances may be linked to ASD rather than to environmental differences between cases and controls. It further underscores the excess of some gut microbiota-derived compounds in ASD, which could have diagnostic value in a network model differentiating the metabolome of autistic and unaffected siblings. Finally, it points toward the existence of a “metabolic autism spectrum” distributed as an endophenotype, with unaffected siblings possibly displaying a metabolic profile intermediate between their autistic siblings and unrelated typically-developing controls.

## 1. Introduction

Autism Spectrum Disorder (ASD) spans a wide range of neurodevelopmental conditions characterized by deficits in social interaction and communication, repetitive behaviors, restricted and unusual interests, rigid adherence to routines, and abnormal sensory processing [1]. Its prevalence rates range from 1/54 children and 1/45 adults in the United States [2,3] to 1/87 children in Italy and 1/102 adults in England [4,5]. ASD displays significant heterogeneity in terms of etiology, symptomatology, developmental trajectory, level of disability and degree of support required for daily living [6]. Autism is caused by neurodevelopmental anomalies established in the prenatal and/or early postnatal period, generally producing relative hypo-connectivity between distant brain areas in the face of local hyperconnectivity [7]. The resulting neural networks show a reduced capacity to integrate information, producing, in addition to the pathognomonic deficit of social cognition, deficits in sensory–motor integration, movement coordination, executive functions and abstract thinking [6]. ASD begins in early childhood, and autistic behaviors are first observed in the majority of children during the second year of postnatal life [6]. The etiology of ASD is very heterogeneous and complex. On the one hand, the existence of a strong genetic component has been conclusively demonstrated: up to approximately 40% of cases appear due to a syndromic form, a single monogenic cause or an oligogenic form involving multiple Copy Number Variants (CNVs) and/or rare germline inherited variants or de novo mutations in epistatic interaction; about 5% of ASD cases are estimated to be due to somatic mutations [8]. On the other hand, the scenario most likely to explain the remaining majority of cases implies gene x environment interactions, whereby a genetic predisposition conferred by common variants and/or rare variants with incomplete penetrance seemingly interacts with an environmental component either directly deranging neurodevelopment or producing an epigenetic and/or splicing dysregulation with functional consequences synergistic to those of the predisposing genetic variants. In addition to environmental factors classically known to be able to cause ASD, such as prenatal exposure to valproic acid and infections with rubella or cytomegalovirus [9], several more common factors have been shown to possibly provide additive or synergistic contributions to ASD, including preterm birth, perinatal hypoxic-ischemic damage, immunological activation during pregnancy, several chemical pollutants, etc. [10,11]. In this area of investigation, increasing interest is being raised by the microbiote, which may produce metabolites able to derange neurodevelopment either directly, as in the case of p-cresol [12,13], or indirectly, for example, by altering the splicing of neurodevelopmentally-relevant genes in the central nervous system [14]. To add another layer of complexity, epigenetic dysregulation in the promoter of genes relevant to neurodevelopment has even been found in the sperm cells of fathers of children with ASD, unexpectedly extending the possibility of gene–environment interactions to parental gametes, involving the generations upstream of the affected child [15]. 

Noticeably, after a first child is diagnosed with ASD, his/her future “baby siblings” will be at higher risk of ASD compared to the above-mentioned approximate 1% population prevalence: ASD recurrence rates in these families are, in fact, estimated on average at 15–25% for male newborns and 5–15% for female newborns [16,17]. Furthermore, first-degree relatives sometimes display minor autistic traits, which are indeed subthreshold relative to a full DSM-5 diagnosis, but indeed witness the existence of a continuum of the autism spectrum in the general population, rather than an “affected/unaffected” dichotomy [18]. Genetics is a logical contributor to familial risk because siblings share 50% of their genome, a lot more than genetically unrelated individuals, but also shared environment may well contribute [19,20], as outlined above, and with the exception of patients carrying known monogenic etiologies, it is generally not possible at the single family level to tease out the relative role of genes and environment. For example, unaffected siblings at times do carry some of the genetic variants that confer ASD vulnerability to their autistic siblings but display only minor or no traits thanks to resilience factors, which may be either genetic or environmental/epigenetic in nature [21]. Importantly, regardless of the cause leading to ASD in every single instance, it is clear that, in multiple ways, unaffected siblings of autistic individuals are not equivalent to unrelated typically-developing controls. The study of families with multiple children, some affected by ASD and others unaffected, can thus provide important clues to the etiology of ASD, above and beyond the study of unrelated samples of children with ASD vs. typical development. This information may also provide clinical benefits because the development of early intensive intervention programs in this last decade and the demonstration of their efficacy in a sizable subgroup of young children displaying autistic behaviors pressures health systems to devise strategies aimed at promoting early and reliable diagnoses. To date, the diagnosis of ASD is still based on clinical observation and would greatly benefit from the identification of objective biological markers for screening, detection, and/or risk estimation, especially in families at increased risk of having a second child diagnosed with ASD. 

Several studies have reported an altered metabolome associated with ASD during childhood, either in blood [22,23] or in urine [24]. However, although some biochemical markers or sets of markers seem promising [25], none has yet been proven robust enough for reliable early intra-family detection. Furthermore, it remains unclear at what point in life biochemical abnormalities of ASD become detectable. Until a few years ago, the use of metabolomics in clinical practice was unthinkable due to its prohibitive costs, but in the last ten years, improved and more affordable technologies have allowed its application in the clinic, with concrete benefits both for the patient and for the containment of healthcare costs. Metabolomics is able to ascertain the presence of biochemical imbalances, which are frequently present in autistic children, mainly involving amino acids, reactive oxidative stress, neurotransmitters, and the microbiota–gut–brain axis, as reviewed by Likhitweerawong et al. [26] and by Garcia-Gutierrez [27]. This approach also allows for the complete elucidation of abnormal biochemical pathways and, in some cases, can offer clues to fundamental abnormalities that may lead to an autistic phenotype in children.

Our initial study [24] involved unrelated individuals, contrasting 30 young autistic children vs. 30 age- and sex-matched typically-developing controls. It unveiled ASD-related imbalances mainly in the tryptophan and purine metabolic pathways. Interestingly, increases were also detected for some tryptophan-related compounds, such as indolyl 3-acetic acid and indolyl lactate, produced exclusively by the gut microbiome. Two subsequent studies extended these initial findings, detecting imbalances in monoaminergic metabolites with increased dopamine and decreased norepinephrine levels [28] in conjunction with decreased levels of vitamins B6, B9 and B12 [29]. The aim of the current study is to replicate and extend these previous findings in same-sex, age-matched pairs of autistic children and typically-developing siblings. Using this within-family paired design, we aim to identify metabolome variations that can be used to discriminate between ASD patients and unaffected siblings, minimizing the influence of confounding factors present in an unpaired design, such as case-control differences in age, gender, diet and exposure to environmental factors, allowing a more reliable identification of the metabolic fingerprint related to the disorder.

## 2. Material and Method

### 2.1. Sample Collection

#### 2.1.1. Subjects Recruitment

Within-family pairs, including one autistic and one unaffected sibling, were selected from a sample of over 300 ASD families on the basis of sex- and age-matching (±2 y). Ultimately 14 pairs were recruited and analyzed, yielding a total of 28 subjects, whose demographic and clinical characteristics are summarized in Table 1. The male-to-female ratio was 3.7:1 (M:F = 11:3), in line with the known male predominance of ASD diagnoses [2,3,4,5]. ASD patients were not receiving any psychopharmacological treatment at the time of urine collection; two patients were taking melatonin (1 mg) at bedtime. In reference to co-occurring conditions, 7/14 (50%) patients had sleep disorders (difficulty falling asleep and/or night awakenings) and 4/14 (28.6%) had chronic constipation. In reference to their diet, two ASD cases were on a gluten-free diet, and one was on a casein-free diet, while the remaining 11 cases were not following any specific diet; three children were taking only semi-liquid foods due to deficits in chewing and swallowing solid foods. The study was approved by the local Ethical Committee (19 June 2017), and in accordance with the Helsinki Declaration, written informed consent was obtained from both parents of each child. Children were diagnosed with ASD based on DSM-5 criteria [1], although DSM-IV subtyping was still performed (Table 1). The clinical diagnosis was confirmed using ADOS-2 [30] and ADI-R [31]; additional psychodiagnostic assessments were performed, as previously described [32]. 

#### 2.1.2. Sample Preparation

First-morning urine samples (10–30 mL) were collected at home by parents using sterile containers un-treated with preservatives and were brought to each clinical center the same morning in wet ice. Urine samples were then aliquoted, frozen, shipped in dry ice, and stored at −80 °C continuously until analysis.

An aliquot of each urine sample was thawed and normalized by urinary specific gravity (see Supplementary Table S1). Each normalized urine sample (Supplementary Table S1) was then added to 1000 μL of a chloroform/methanol/water (1:3:1 ratio) solvent mixture stored at −20 °C. The tubes were mixed for 30 min and subsequently centrifuged at 1000× *g* for 1 min at 4 °C, before being transferred to −20 °C for 2–8 h. The solutions were then centrifuged for 15 min at 15,000× *g* and were dried to obtain visible pellets. Finally, the dried samples were re-suspended in 0.1 mL of water, 5% formic acid and transferred to glass autosampler vials for LC/MS analysis. Quality controls (QCs) were obtained from a pooled mixture of 10 μL aliquots of all urine samples and were analyzed every 5 samples.

### 2.2. Metabolomic Analysis and Data Processing

Twenty microliters of extracted supernatant samples was injected into an ultrahigh-performance liquid chromatography (UHPLC) system (Ultimate 3000, Thermo, Rockford, IL, USA) and run on a positive mode: samples were loaded onto a Reprosil C18 column (2.0 mm × 150 mm, 2.5 μm-Dr. Maisch, Ammerbuch-Entringen, Germany) for metabolite separation. For positive ion mode (+) MS analyses, a 0–100% linear gradient of solvent A (ddH_2_O, 0.1% formic acid) to B (acetonitrile, 0.1% formic acid) was employed over 20 min, returning to 100% A in 2 min and holding solvent A for a 1-min post time hold. Acetonitrile, formic acid, and HPLC-grade water and standards (≥98% chemical purity) were purchased from Sigma Aldrich. Chromatographic separations were made at a column temperature of 30 °C and a flow rate of 0.2 mL/min. The UHPLC system was coupled online with a Q Exactive mass spectrometer (Thermo) scanning in full MS mode (2 μ scans) at a resolution of 70,000 in the 67 to 1000 *m*/*z* range, a target of 1106 ions and a maximum ion injection time (IT) of 35 ms with 3.8 kV spray voltage, 40 sheath gas and 25 auxiliary gas. LC-MS/MS analysis of each sample was performed in order to achieve the mass fragmentation spectra. In this method, during the chromatographic run, both full scan and MS2 spectra of the three most intense ions of each full scan were acquired. The resolving power for MS2 scans was 7000. Product ions were generated in the LTQ trap at collision energy 30 eV using an isolation width of 2 Da.

Calibration was performed before each analysis against positive or negative ion mode calibration mixes (Pierce, Thermo Fisher, Rockford, IL, USA) to ensure the error of the intact mass within the sub ppm range.

Replicates were exported as .mzXML files and processed through MAVEN.8.1. Mass spectrometry chromatograms were created for peak alignment, matching and comparison of parent and fragment ions with tentative metabolite identification (within a 2 p.p.m. mass-deviation range between the observed and expected results against an imported KEGG database). Data were at first normalized by sum, logarithmic transformation, and auto-scaling, according to Pareto. Univariate statistical analysis included the non-parametric Mann–Whitney U test and the Spearman’s rho (ρ) correlation test. The non-parametric Mann–Whitney U test evaluated differences in urine metabolite levels between groups; *p* < 0.05 was considered statistically significant.

Fold change analysis was performed on the entire metabolomics data set using the MetaboAnalyst 5.0 software (https://www.metaboanalyst.ca/docs/Publications.xhtml, accessed on 15 August 2022). Before the analysis, raw data were normalized by median and autoscaling in order to increase the importance of low-abundance ions without significant amplification of noise. The purpose of fold change (FC) analysis was to compare absolute value change between two group averages and find some features that are changed consistently (i.e., upregulated or down-regulated) between two groups. MetPA was used to construct and analyze metabolic pathways; the species was set to the human database, and the numbers of the potential metabolites were entered for this pathway analysis. Using topological analysis, the cutoff value of the metabolic pathway influence was set to 0.2, and pathways with a value greater than 1 were selected as potential key metabolic pathways. 

### 2.3. Calculation of Concentration Ratios of Selected Urinary Metabolites to Specific Gravity

Urinary specific gravity was measured by refractometry following centrifugation at 13,000× *g* for 10 min, using a digital refractometer (Euromex Clinical Digital Refractometer RD.5712, NL) previously calibrated with LC-MS grade water.

## 3. Results

### Metabolic Profiling Using Untargeted Metabolomics in LC-MS Platform

**Figure 1:**
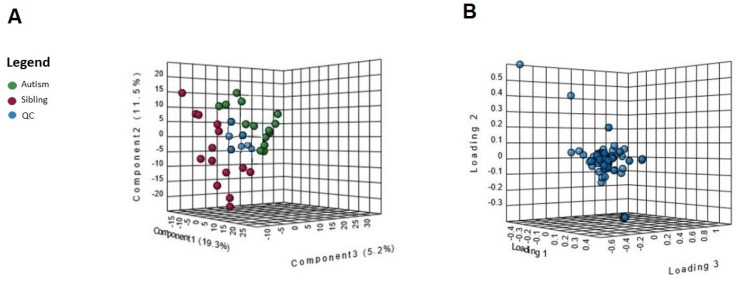
(**A**) Multivariate statistical analysis based on untargeted metabolite profile data derived from urine samples of ASD patients (red), typically-developing siblings (green) and quality controls (blue). Principal component analysis (PCA) in 3D based on normalized and mean-centered data of the 28 samples after outlier removal. Each sphere represents one sample. Axes are principal components 1 (x) and 2 (y), explaining 19.3% and 11.5% of the variation in the data, respectively. (**B**) Loading plots of the first two principal components for the platform metabolites.

**Figure 2:**
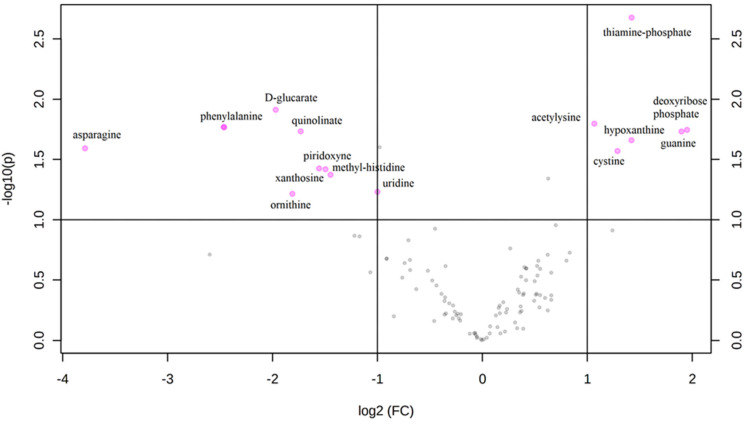
Volcano plot: each point on the volcano plot was based on both *p*-value and fold-change values, and in this study, these two values were set at 0.05 and 2.0, respectively. The points that satisfy the condition *p* < 0.05 and fold change > 2.0 appear in pink color and are marker candidates, whereas the others appear in gray and do not reach significance. On the *x*-axis, log2 (FC) is positive when ASD > SIB and negative when ASD < SIB, so pink dots on the right side of the figure represent metabolites significantly upregulated in autistic patients, and the pink dots on the left side represent the down-regulated metabolites. The *x*-axis corresponds to log2 (fold change), and the *y*-axis corresponds to –log10 (*p*-value).

An untargeted metabolomics analysis was performed, and more than 1000 peaks per sample were referred to the KEGG database; among them, 256 metabolites have been analyzed more precisely and identified. The Principal Component Analysis (PCA) score plot derived from the untargeted metabolomics data indicated that the ASD group and their typically-developing siblings could be separated and that the first and second principal components (PC1 and PC2) explain 19.3% and 11.5% of the variance, respectively (Figure 1A). Starting from the loading plots displayed in Figure 1B, where each data point represents the entire metabolome of a single individual and some data points may be superimposed to each other, various discriminating metabolites could then be identified as responsible for the clear separation between ASD children and their typically-developing siblings by using Volcano plot analysis (Figure 2).

This univariate analysis identified a significant accumulation of specific metabolites; six of them are found in excess among autistic children (thiamine-phosphate, deoxyribose-phosphate, hypoxantine, guanine, cystine and acetylysine), while nine others were overexpressed in siblings (D-glucarate, phenylalanine, quinolinate, asparagine, piridoxyne, methyl-histidine, xanthosine, uridine, and ornithine).

**Figure 3:**
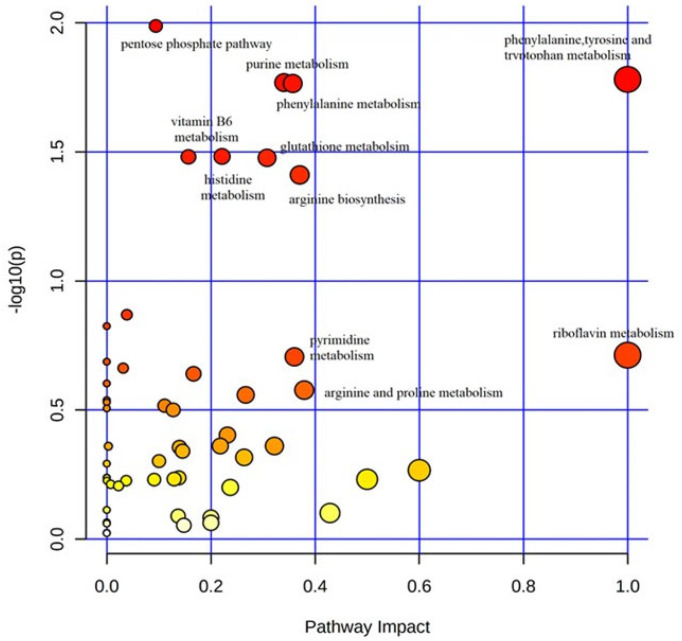
Metabolic pathway analysis plot. Color intensity (white to red) reflects increasing statistical significance, while circle diameter covaries with pathway impact. The graph was obtained by plotting on the *y*-axis the −log of *p* values from the pathway enrichment analysis and on the *x*-axis the pathway impact values derived from the pathway topology analysis. The metabolic pathways displaying the largest differences in ASD vs. typically-developing siblings mainly corresponded to “phenylalanine-tyrosine-and-tryptophan metabolism”, “phenylalanine metabolism”, “purine metabolism”, and “glutathione metabolism”.

The KEGG pathway analysis of differentially abundant metabolites was performed by MetaboAnalyst 5.0 to identify the disturbed metabolic pathways in autistic children compared to their typically-developing sibling. Results of the “metabolome overview” obtained through metabolic pathway analysis (MetPA) displayed in Figure 3 reveal that the most perturbed metabolic pathway in ASD mainly corresponded to “phenylalanine-tyrosine-and-tryptophan metabolism”, followed by “phenylalanine metabolism”, “purine metabolism” and “glutathione metabolism”. These metabolic pathways will thus be discussed in greater detail. 

**Figure 4:**
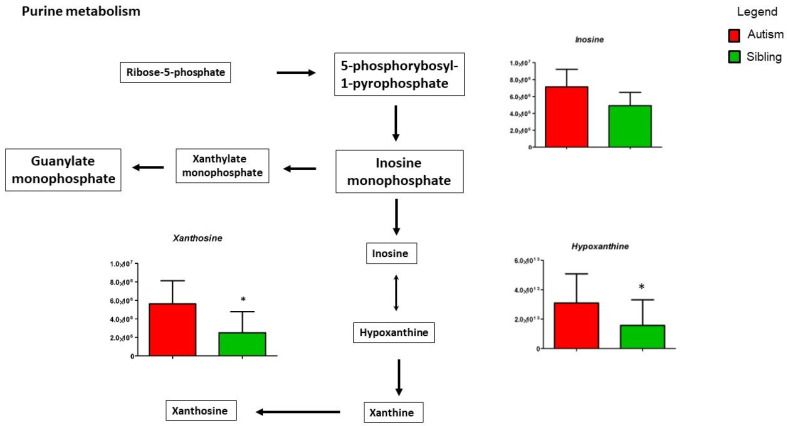
Intermediates of purine pathway are presented as the differences between autistic subjects in red and typically-developing siblings in green. The columns represent the mean ± SD (n = 14) of each metabolite concentration. * *p* < 0.05.

Given the recurrent involvement of tryptophan and purine metabolisms in autism spectrum disorder [24] and given the relevance of tryptophan- and purine-derived compounds in many neural functions, we initially focussed our attention on these metabolisms (Figure 4).

Although Figure 4 shows all the intermediates belonging to the tryptophan pathway found using mass spectrometry analysis, only three of them show a statistically significant variation. We notice an increase in xanthurenic acid and quinolinic acid and lower serotonin levels. In the brain, quinolinic acid acts as a gliotoxin, proinflammatory mediator, and pro-oxidant molecule, boosting oxidative stress by stimulating microglia to release large amounts of NO and superoxide; in addition, it exerts excitotoxic effects by acting as an N-methyl-D-aspartate (NMDA) receptor agonist, stimulating glutamate release, blocking glutamate reuptake into astrocytes, and reducing the activity of glutamine synthase [33,34]. 

**Figure 5:**
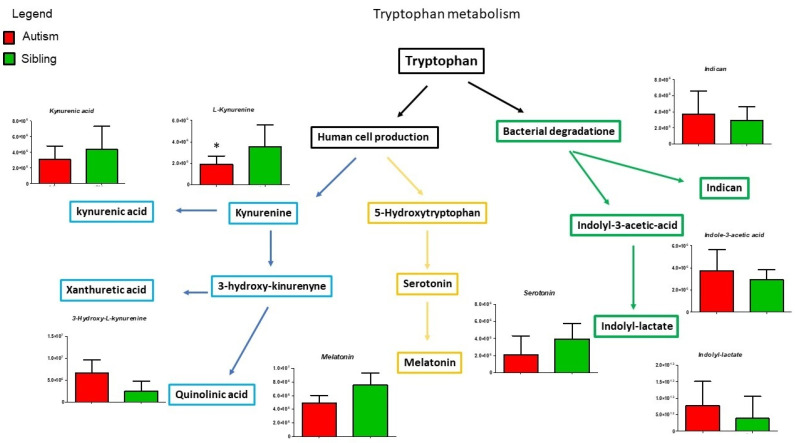
Intermediates of purine metabolism are presented as the differences between autistic subjects in red and typically-developing siblings in green. Inosine, Xanthosine and Hypoxanthine appear to be increased in autistic patients in the urine. The columns represent the mean ± SD (n = 14) of each metabolite concentration. * *p* < 0.05.

Figure 5 shows purine metabolism, in particular, hypoxanthine and xanthosine, significantly increased in ASD patients compared to typically-developing siblings, while inosine shows a non-significant decrease. Importantly, the release of these purine metabolites triggers the “cell danger metabolic response” involving mitochondrial dysfunction, microglial activation and neuroinflammation [35].

**Figure 6:**
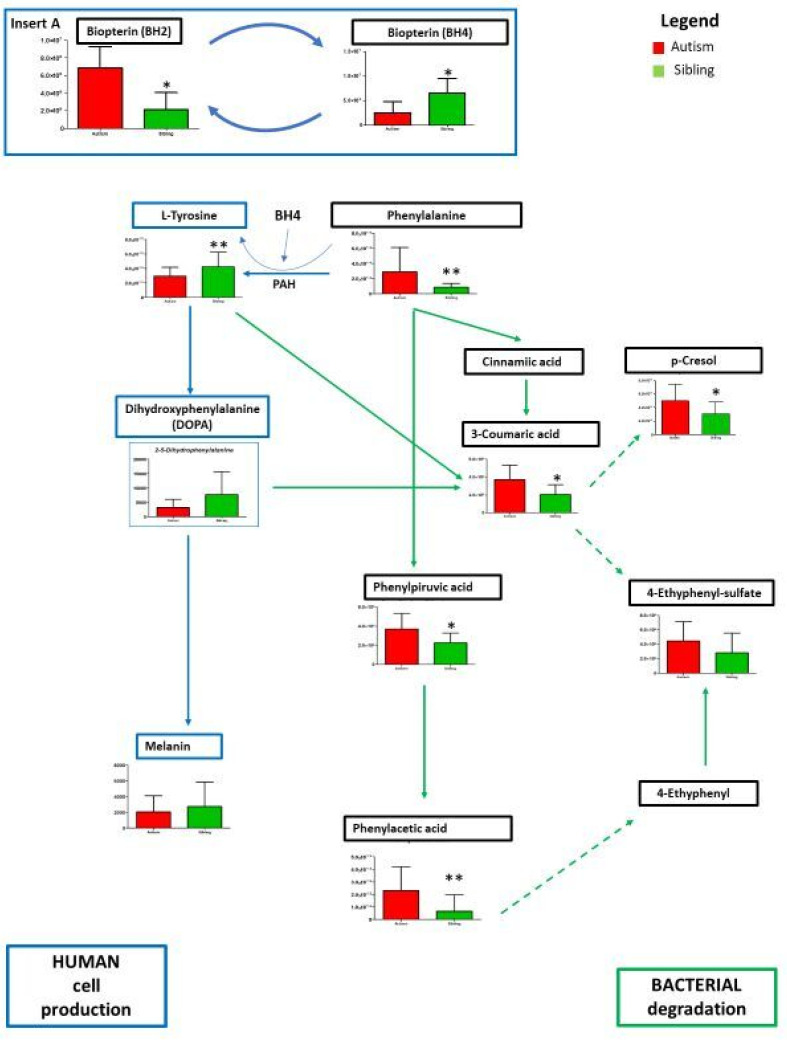
Intermediates of the phenylalanine/L-tyrosine pathway by human cells and gut bacteria, leading also to the formation of 4-ethylphenol sulfate (4-EPS) and p-cresol. are presented as the differences between autistic subjects in red and typically-developing siblings in green. The columns represent the mean ± SD (n = 14) of each metabolite concentration. * *p* < 0.05, ** *p* < 0.01, etabolites derived from bacterial degradation are boxed in green, metabolites from human cell production in blue. Solid lines represent established reactions, dotted lines represent presumed reactions taking place.

An in-depth analysis concerned the metabolism of phenylalanine due to its significant variation between paired ASD and “typically-developing siblings”. In our results (Figure 6), lower levels of tyrosine are observed in the urine of autistic children compared to matched typically-developing siblings, whereas higher contents of phenylalanine phenylethylamine, phenylpyruvic and phenylacetic acid have been found. Noticeably, decreases in tyrosine and increases in phenylalanine are paralleled by reduced levels of tetrahydrobiopterin (BH4) and increased levels of dihydrobiopterin (BH2) in ASD compared to unaffected siblings (Figure 6, insert A). BH4 is well-known to play a critical role in the conversion of phenylalanine to tyrosine (see Discussion).

On the other hand, phenylpyruvic and phenylacetic acid are products of phenylalanine degradation from fermentable substrates by the intestinal gut microbiota. Figure 6 represents an overview of gut and host-microbial metabolism involved in the production of p-cresol [11,12,36,37] and of 4-ethylphenyl-sulfate (4EPS) [14,38]. These two metabolites derived from gut bacteria are gaining more attention in ASD research due to their neurochemical and behavioral effects [13,14,37,38,39]. In this sample, p-cresol is significantly increased in ASD over siblings, whereas 4-EPS shows a non-significant trend.

## 4. Discussion

The present study, by using a multivariate statistical approach, describes significant urinary metabolomic differences between young children with idiopathic ASD and their typically-developing siblings, which confirms and expands previous observations by our group. Since the urine content reflects what is happening in the whole organism, with our metabolomics analysis, we are closer to taking into account the entire patient with a systemic view. By studying and quantifying the metabolites present in biological fluids, metabolomics offers an instantaneous view of the system, providing useful information for interpreting the processes in the analyzed organism. The metabolites, or the small molecules, can be considered as the final product of gene expression and protein activity, therefore determining the biochemical phenotype of a biological organism. Through metabolomics, indeed, it is possible to provide researchers and clinicians with the most up-to-date information on possible biomarkers that can help them understand how to act on therapeutic strategies for patients. At present, routine metabolic testing has been recommended for patients with autism only on the basis of physical examination features or historical details such as seizures, developmental regression, or acidosis, provided the child has passed the relevant state-mandated newborn screening [26].

We previously demonstrated that untargeted metabolomics may be used to identify many of the diagnostic and even secondary changes in downstream analytes by contrasting young children with idiopathic ASD and unrelated typically-developing controls [24,28,29]. Applying this case-control design involving unrelated individuals, we indeed found an imbalance in several compounds belonging mainly to the metabolism of purines, amino acids, and tryptophan pathways, as well as compounds derived from the intestinal flora and reduced levels of vitamins B6, B12 and folic acid [24,28,29]. Considering that metabolomic approaches identify perturbations in metabolic pathways that are determined by genetic and environmental factors [40] and that typically-developing siblings of autistic patients can be considered a population that is intrinsically different from non-genetically-related typically developing controls, as outlined in the Introduction, in this study we focused our attention on contrasting autistic patients with their typically-developing siblings using an intra-family design. Furthermore, contrasting autistic children with their unaffected siblings reduces the probability of potential bias, linked to differential exposure to environmental factors depending on the residential area, for example, and minimizes, though it does not eliminate, the possible bias introduced by differential feeding habits. Noticeably, here we find alterations in the same tryptophan pathways and purines metabolism whose trends are superimposable to those observed, contrasting ASD cases and typically-developing controls [24], but metabolite concentrations consistently differ to a lesser extent between ASD cases and their matched unaffected siblings [24]. In this regard, ASD-associated metabolic profiles may seemingly represent “endophenotypes”, not only associated with ASD as “biomarkers” but also displaying intermediate phenotypes in first-degree relatives due to familial genetic contributions and/or to intrafamilial shared environment [41]. 

Indeed, lower levels of tyrosine and DOPA in the urine of autistic children compared to typically-developing siblings were observed and perhaps the most striking result to emerge from our data consists in the elevated urine content of phenylalanine (Figure 6). The essential amino acid phenylalanine is a metabolic precursor to tyrosine (Tyr) via phenylalanine hydroxylase (PAH) activity in the liver; a reduction in tyrosine and DOPA in the urine of young children with idiopathic ASD, led us to hypothesize a deficiency in PAH activity or in its cofactor tetrahydrobiopterin (BH4). Our results indeed support a reduction in BH4 as a significant contributor to reduced PAH activity and to the tyr/phe imbalance described here. Nonetheless, the complexity of these metabolic pathways does not allow us to exclude that, in addition to contributions by reduced BH4 levels, PAH activity may not be decreased.

Phenylalanine and tyrosine are metabolized in similar ways by gut bacteria, leading to the formation of 4-ethylphenol and p-cresol, which can then be conjugated by the host into 4-ethylphenyl sulfate and p-cresyl sulfate, respectively. Both these compounds originate mainly from bacterial fermentation of the amino acid phenylalanine and, to a smaller extent, from amino acid tyrosine. Tyrosine and phenylalanine can undergo reductive as well as oxidative metabolism by intestinal bacteria [42]. From Figure 6, it emerges that p cresol can be derived from the catabolism of both tyrosine and phenylalanine; however, the presence of high amounts of 4-hydroxyphenylpyruvic acid, phenylpyruvic acid and phenylacetic acid, which are derived exclusively from the catabolism of phenylalanine, supports the existence of a greater concentration of phenylalanine in the urine of autistic patients contrasted with typically-developing siblings. Phenylpyruvic, phenylacetic acid as bacteria degradation and fermentable substrates for the gut microbiota undergo intense proteolysis into amino acids, and colonic amino acids may be further metabolized by the gut microbiota. Moreover, 4-ethylphenyl sulfate, together with p-cresol and p-cresyl sulfate, have been associated with ASD in both young affected children [12,22,24,43,44,45] and cellular/animal models [13,37,38,39,46]. Importantly, p-cresol has been recently shown to impair neural differentiation and decrease both neurites outgrowth and synaptogenesis in neuronal cell lines [13]. p-Cresol and 4-ethylphenol are produced through aromatic amino acid fermentation by a range of commensal bacteria, most notably bacteria from the Clostridioides genus, which are among the dysregulated bacteria frequently detected in ASD patients [46]. In addition to host metabolism, there is ample evidence that the gut microbiota is actively involved in aromatic amino acid (AAA) metabolism. When the activity of the enzyme PAH is reduced, the amino acid phenylalanine accumulates and is converted into phenylpyruvic acid (phenylpyruvate) by the gut microbiome, which also transforms AAAs into numerous metabolites that may regulate immune, metabolic, and neuronal responses at local and distant sites. This chemical dialogue between host cells and the gut microbiome is shaped by environmental cues and may become dysregulated in gastrointestinal and systemic diseases. These biochemical changes are consistent with some of the known abnormalities of gut microbiota found in autistic individuals. From this point of view, environmental factors could play a relevant role in clinical features, and among them, the overgrowth of unusual gut microbial species in a group of autistic patients is of great interest, as reported in several recent studies [47,48,49,50]. The possibility that alterations in the gut may be important in the pathophysiology of human central nervous system disorders is now increasingly appreciated [51]. As a typical mechanism of host–microbe communication, the gut microbiota produces thousands of small molecules and metabolites that accumulate in the gastrointestinal system or reach distant organs. Well documented is the toxic retention solutes by p-cresol, when they reach high concentrations in uremic patients [52,53,54]. 

The present study has at least two main limitations. First, the sample size is relatively small due to the difficulty in recruiting families with affected and unaffected sibling pairs tightly matched by sex and age. Secondly, while our patient sample is well-characterized and in all regards fits with the prevalence rates of behavioral symptoms and co-morbid conditions, such as sleep disturbances and gastrointestinal issues, as previously reported in ASD [55,56], unaffected siblings have not been formally assessed for behavioral disorders and medical issues. Their “typical development” is defined only based on parental reports and a brief interview with our medical personnel. Despite these limitations, confidence in the reliability of the present findings is strengthened by our replication of previous results [24] obtained using two non-overlapping ASD samples contrasted with two entirely distinct control samples, namely 30 unrelated controls in Gevi et al. (2016) [24] and 14 unaffected siblings in this study.

## 5. Conclusions

The analysis of urine by applying metabolomic approaches appears to be a promising avenue to learn more about ASD and even correctly distinguish autistic from typically-developing siblings within families at increased risk of recurrence. In this study, by contrasting young children with idiopathic ASD and their typically-developing siblings, we confirmed our previous results obtained contrasting ASD children with unrelated typically developing controls, namely an alteration of purine and tryptophan metabolism. The present data, furthermore, distinguish between ASD and typically-developing siblings based on excessive urinary concentrations of phenylalanine, possibly due to both a malfunction of the PAH enzyme and reduced amounts of its cofactor BH4. Decreased phenylalanine transformation into tyrosine would be predicted to yield increased blood concentrations and, therefore, greater phenylalanine excretion in the urines. However, measurements of phenylalanine plasma levels in ASD vs. typically developing controls provide contrasting results, with ASD < CON in most studies [57,58,59], ASD > CON in Aldred et al. (2003) [60], and ASD = CON, but phe/tyr ratio ASD > CON, in Bala et al. (2016) [61]. This sampling variability again underscores the importance of replicating our previous results [24]. Finally, collectively, our studies provide converging evidence that altered metabolomic profiles in ASD may represent an endophenotype, reflecting the existence of a “metabolic autistic spectrum” whereby unaffected siblings may represent an intermediate phenotype between autistic siblings and typically developing controls. Whether and to what extent this depends on shared genetic or environmental underpinnings, including partially shared gut microbiota composition between affected and unaffected siblings, remains to be elucidated. Contrasting well-matched ASD-unaffected siblings and unrelated control trios in the same study will be necessary to address these important questions.

## Figures and Tables

**Table 1:** Demographic characteristics of the sample (N = 14 ASD and 14 typically-developing siblings) and main clinical features of the 14 children with ASD. * One ASD patient with intellectual disability was not testable. Abbreviations: IQ, Intellectual Quotient; PDD-NOS, Pervasive Developmental Disorder—Not Otherwise Specified; SEM, Standard Error of the Mean; VABS, Vineland Adaptive Behavior Scales.

		N	Mean/Median (Range) or %
Age in yrs (mean± SEM)	-ASD -Unaffected siblings	14 14	7.06 *±* 0.96 (3.2–15.6) 6.68 *±* 1.28 (1.0–14.0)
Gender:	Male	11 pairs	78.6%
	Female	3 pairs	21.4%
	M/F ratio		3.7:1
I.Q.	Mean ± SEM	13 *	64.7 *±* 6.78 (30–104)
	>70	7	50.0%
	≤70	7	50.0%
DSM-IV Diagnosis:	Autistic Disorder	10	71.5%
	Asperger Syndrome	1	7.1%
	PDD-NOS	3	21.4%
Level of Expressive Language	Sentences	3	21.4%
	Words	7	50.0%
	Non-verbal	4	28.6%
Median VABS Scores:			
Communication		12	73.5 (31–115)
Daily Living Skills		12	78.5 (48–113)
Socialization		12	73.0 (55–116)
Motor Skills		8	87.5 (56–111)
Composite		12	73.5 (47–115)

## Data Availability

The data presented in this study are available on reasonable request to the corresponding author. The data are not publicly available due to the protocol approved by the Ethical Committees.

## Supplementary Materials

The following supporting information can be downloaded at: https://www.mdpi.com/article/10.3390/metabo12090797/s1, Table S1: Demographic characteristics of the sample (N = 14 ASD and 14 unaffected siblings) and main clinical features of the 14 children with ASD.
