# Supplementary material for: Urinary Untargeted Metabolic Profile Differentiates Children with Autism from Their Unaffected Siblings
Source: Metabolites. 2022 Aug 26;12(9):797. doi: 10.3390/metabo12090797 (PMC9503174; doi:10.3390/metabo12090797)
Supplement: Supplementary file 1 [file metabolites-12-00797-s001.zip › metabolites-1831520-supplementary.pdf]

Table S1. Demographic characteristics of the sample (N=14 ASD and 14 unaffected siblings) and main clinical features of the 14 children with ASD.

		N	Mean/Median (range) or %
Age in yrs (mean± SEM)	*- ASD*	14	7.06 ± 0.96 (3.2-15.6)
	- Unaffected siblings	14	6.68 ± 1.28 (1.0-14.0)
Gender:	*Male*	11 pairs	78.6%
	Female	3 pairs	21.4%
	M/F ratio		3.7 : 1
I.Q.	*mean± SEM*	13*	64.7± 6.78 (30-104)
	>70	7	50.0%
	≤ 70	7	50.0%
DSM-IV Diagnosis:	*Autistic Disorder*	10	71.5%
	Asperger Syndrome	1	7.1%
	PDD-NOS	3	21.4%
Level of expressive language	*Sentences*	3	21.4%
	Words	7	50.0%
	Non-verbal	4	28.6%
Median VABS scores:			
Communication		12	73.5 (31-115)
Daily living skills		12	78.5 (48-113)
Socialization		12	73.0 (55-116)
Motor skills		8	87.5 (56-111)
Composite		12	73.5 (47-115)

*One ASD patient with intellectual disability was not testable.

Abbreviations: IQ. intellectual quotient; PDD-NOS. Pervasive Developmental Disorder – Not Otherwise Specified; SEM. standard error of the mean; VABS. Vineland Adaptive Behavior Scales.
